# Treatise on the Sympathetic Relation between the Stomach and the Brain, and, Throughout, between the Digestive and the Nervous Systems, in the Causation and Cure of Diseases. With an Appendix Containing a Few Observations on Certain Points Connected with the Treatment of Chronic Disease, and Its Attendant Debility

**Published:** 1841-04

**Authors:** 


Art. II.—*Treatise on the Sympathetic Relation between the Stomach and the Brain, and, throughout, between the Digestive and the Nervous Systems, in the Causation and Cure of Diseases. With an Appendix containing a few Observations on certain points connected with the Treatment of Chronic Disease, and its attendant Debility.* By Charles Wightman, m.d., Licentiate of the Royal College of Physicians of London, and Resident Physician in Newcastle-upon-Tyne. —*London*, 1840. 12mo, pp. 192.

This is truly a big title for a book so little! We are told in the Preface that not only no specific treatise upon the subject, has hitherto existed in the English language, but that the only bibliographical notices which Dr. Wightman can discover in reference to it, are of two unpurchaseable Dissertations published towards the end of the last century, by Drs. Rahn and Veegens. " If then," continues the Resident Physician, " there should be any similarity between them and my own treatise, it must be regarded as a circumstance purely accidental." . . . "But I wish it to be distinctly known, that to no author who has written *expressly* upon the subject, have I been indebted for a single remark connected therewith." Nevertheless we will venture to affirm, that there is nothing of any value in Dr. Wightman's book with which the well-educated medical practitioner is not perfectly familiar; and we can scarcely help placing it, therefore, in the second of the classes we mentioned in the last notice; to which, indeed, the superfluous minuteness of the title-page would have predisposed us, before reading it, to refer it.

There is plenty of room for a really philosophical treatise which shall afford a satisfactory elucidation of those remarkable sympathetic relations between the nervous centres and the digestive system, which the phenomena of disease so abundantly reveal; but such an explanation must be founded upon the soundest physiological inductions, and not (as most part of Dr. Wightman's unfortunately is,) upon doctrines that have not stood the test of experience. As Dr. Wightman expresses himself as anxious for any additional observations of interest, we may mention a case which came under our own notice, in which a gentleman, predisposed to derangement of the nervous system by his excessive mental activity, was one day attacked, after eating a rather full luncheon of somewhat indigestible food, with a sudden and general but incomplete loss of muscular power, like that which takes place in partial syncope, but without any corresponding depression of the mental energies. What muscular force remained was principally exercised in a tendency to turn round to the right side, like that stated by Magendie to follow division of the crus cerebelli, and other injuries of the base of the brain. The stomach was speedily relieved by spontaneous vomiting; and the patient completely recovered in a few hours.

Although we have spoken with disapprobation of the manner in which Dr. Wightman's treatise is put forth, we must do the author the justice to say that we think it may be useful to students and young practitioners, in drawing their attention to the intricacies of diagnosis in the class of cases to which it refers.

